# Efficacy of Huoxue Tongqiao Decoction combined with aniracetam in treating post-stroke cognitive impairment in elderly patients with ischemic stroke and its effects on serum levels of superoxide dismutase, glutathione peroxidase and nitric oxide

**DOI:** 10.12669/pjms.42.1.12740

**Published:** 2026-01

**Authors:** Jinjin Xu, Xue Bai, Hui Wang, Jiangzhe Li, Pan Cai

**Affiliations:** 1 Jinjin Xu Department of Geriatric, Baoding NO.1 Central Hospital, Baoding 071000, Hebei, China; 2 Xue Bai Department of Physical Examination, Baoding NO.1 Central Hospital, Baoding 071000, Hebei, China; 3 Hui Wang Department of Urological, Baoding NO.1 Central Hospital, Baoding 071000, Hebei, China; 4 Jiangzhe Li Western Medicine Pharmacy Static Dispensing Center, Baoding NO.1 Central Hospital, Baoding 071000, Hebei, China; 5 Pan Cai Department of Rheumatology and Immunology, Baoding NO.1 Central Hospital, Baoding 071000, Hebei, China

**Keywords:** Aniracetam, Cognitive impairment, Cerebral perfusion, Glutathione peroxidase, Huoxue tongqiao decoction, Nitric oxide, Senile ischemic stroke, Superoxide dismutase

## Abstract

**Objectives:**

To evaluate the clinical efficacy of Huoxue Tongqiao (blood-invigorating and orifice-unblocking) Decoction (HXTQD) combined with aniracetam in the treatment of post-ischemic stroke cognitive impairment (PISCI) in elderly patients and its effects on related serum biochemical markers.

**Methodology:**

This was a retrospective study. A total of 80 elderly patients with PSCI admitted to Baoding No.1 Central Hospital between January 2022 to December 2024 were enrolled and randomly assigned to either the control group or the observation group using a random number table. All patients received standard internal medicine management. The control group was treated with aniracetam, while the observation group was orally administered HXTQD in combination with aniracetam. TCM syndrome, MoCA, MMSE, ADL scores, cerebral hemodynamic parameters and serum biochemical markers were assessed before and after treatment. The overall response rate was compared between the two groups.

**Results:**

After treatment, both groups showed significant improvements in MoCA, MMSE and ADL scores, Vm and serum levels of SOD, GSH-Px and NO(all P< 0.05), with the observation group demonstrating significantly greater improvements than the control group (P< 0.05). TCM syndrome scores, RI and PI decreased in both groups, with more pronounced reductions noted in the observation group (P< 0.05, respectively). The ORR in the observation group was significantly higher than in the control group (P< 0.05). No adverse reactions were reported in either group.

**Conclusion:**

The combination of HXTQD and aniracetam can significantly improve cognitive function and cerebral perfusion in elderly patients with post-ischemic stroke cognitive impairment, potentially by regulating serum SOD, GSH-Px and NO levels.

## INTRODUCTION

Ischemic stroke, also known as cerebral infarction, refers to localized ischemic necrosis or softening of brain tissue caused by insufficient cerebral blood flow due to arterial stenosis or occlusion. In recent years, the global population structure has undergone significant changes with the rising proportion of elderly individuals. As a result, cerebrovascular diseases like ischemic stroke have become major health threats, severely affecting the physical and mental well-being and even the survival of older adults. Post-stroke cognitive impairment (PSCI) refers to a complication or sequela that persists for at least six months following the onset of stroke, characterized primarily by deficits in one or more cognitive domains such as memory, perception, or orientation. PSCI often impairs patients’ ability to perform activities of daily living and increases the risk of psychiatric disorders and mortality.^1^

Currently, there is no specific treatment for PSCI in conventional Western medicine. Clinical management mainly focuses on controlling risk factors, preventing recurrent strokes and improving cognitive function, with therapeutic strategies aimed at protecting neuronal cells, enhancing excitatory neurotransmitter activity and improving cerebral circulation. However, most Western pharmacological agents offer only symptomatic relief with limited efficacy in improving cognitive deficits and are often associated with well-documented adverse effects. In contrast, traditional Chinese medicine (TCM) offers distinct advantages in the treatment of post-ischemic stroke cognitive impairment (PISCI).

TCM interventions can rapidly alleviate clinical symptoms, stabilize disease progression, improve patient prognosis and are generally associated with fewer adverse effects, higher safety profiles and better patient acceptance.^2^ Drawing on classical TCM theories and personal clinical experience, the authors view the pathogenesis of PISCI as a combination of deficiency and excess: deficiency primarily involves insufficiency of liver and kidney *qi* and blood, while excess is characterized by the interlocking of phlegm and blood stasis, a pattern that persists throughout the course of the disease. On this basis, Huoxue Tongqiao Decoction (HXTQD) was formulated to invigorate blood, tonify qi, resolve phlegm and unblock the orifices. This formula aligns with the pathophysiological features of the intertwined phlegm and blood stasis syndrome commonly observed in elderly patients with PISCI. In this study, the efficacy of HXTQD combined with the Western medication aniracetam was evaluated by monitoring changes in Montreal Cognitive Assessment (MoCA) and Mini-Mental State Examination (MMSE) scores, cerebral blood flow parameters and related serum biochemical markers before and after treatment.

## METHODOLOGY

This was a retrospective study. A total of 80 elderly patients diagnosed with PISCI and treated at Baoding No.1 Central Hospital between January 2022 and December 2024 were enrolled in this study. Using a random number table, the patients were assigned to either the control group or the observation group (n = 40 per group). Patient data including the medical data of veterans from Baoding No.1 Central Hospital were selected information and management system, collected their various information of all patients. Both groups underwent continuous treatment for three months.

### Ethical Approval:

The study was approved by the Institutional Ethics Committee of Baoding No.1 Central Hospital (No.: [2024]53; date: May 8, 2024) and written informed consent was obtained from all participants and their families.

### Inclusion criteria:

- Meeting the Western diagnostic criteria for PISCI.
- Meeting the TCM diagnostic criteria for intertwined phlegm and blood stasis syndrome.
- Aged 60-75 years.
- Disease duration between three and six months.
- Clear consciousness.
- Written informed consent.

### Exclusion criteria:

- History of dementia or cognitive impairment prior to the onset of ischemic stroke.
- Presence of severe dysfunction in vital organs such as the heart, liver, or kidneys.
- Coexisting neurological disorders such as meningitis or brain tumors.
- Inability to cooperate with treatment due to aphasia, or significant visual or hearing impairments.
- Psychiatric disorders.
- Multiple poorly controlled comorbidities.
- Coagulation disorders.
- Use of cognitive-enhancing medications within the past three months.
- Known allergy to any of the study medications.
- With poor compliance and incomplete treatment.

In the control group, there were 24 males and 16 females, aged 60-75 years with a mean age of (67.18 ± 2.52) years. The disease duration ranged from three to six months, with a mean duration of (4.89 ± 0.41) months. In the observation group, there were 25 males and 15 females, aged 60-75 years with a mean age of (67.20 ± 2.50) years. The disease duration ranged from three to six months, with a mean duration of (4.90 ± 0.40) months. There were no statistically significant differences in baseline characteristics (age, sex, disease duration) between the two groups (all *P* > 0.05).

### Diagnostic criteria:

In the Western medicine framework, diagnosis was established according to the Expert Consensus on Post-stroke Cognitive Impairment Management 2021^3^ and required fulfillment of the following criteria:

- Evidence of ischemic stroke based on clinical manifestations and neuroimaging findings;
- Cognitive impairment reported by the patient or identified through clinical assessment following ischemic stroke, with confirmed impairment in one or more cognitive domains based on neuropsychological testing (MMSE score ≤26 or MoCA score <26);
- Cognitive decline occurring after the ischemic stroke and persisting for three to six months;

Exclusion of non-ischemic causes of cognitive impairment. TCM diagnosis was based on the Guiding Principles for Clinical Research of New Chinese Medicines and the disease falls into the category of intertwined phlegm and blood stasis syndrome^4^. This pattern is characterized by the following signs and symptoms:

### Primary symptoms:

cognitive decline, memory impairment, head heaviness, headache, excessive sputum, poor appetite, cyanotic lips;

### Secondary symptoms:

heaviness of limbs, obesity, nausea, dull facial complexion; (3) Tongue and pulse: enlarged tongue, dark or purplish tongue body with white, greasy coating, petechiae or ecchymoses; deep, slow, or slippery pulse.

### Treatment methods:

Both groups received standard internal medicine management, including nutritional counseling, smoking and alcohol cessation and proactive control of risk factors such as hyperglycemia and hypertension, along with appropriate management of underlying conditions. All patients also received secondary prophylaxis for ischemic stroke using standard Western medications:

Atorvastatin calcium tablets (20 mg; Approval No. H20051408; Viatris Pharmaceuticals (Dalian) Co., Ltd.), 20 mg once daily at night; (2) Clopidogrel bisulfate tablets (75 mg; Approval No. J20180029; Sanofi (Hangzhou) Pharmaceuticals Co., Ltd.), 75 mg once daily at night. In addition to the above, the control group was treated with aniracetam (50 mg; Approval No. H20000548; Yabao Pharmaceutical Group Co., Ltd.). In addition to the above, the control group was treated with aniracetam (50 mg; Approval No. H20000548; Yabao Pharmaceutical Group Co., Ltd.). Patients under 70 years of age received 200 mg per dose, three times daily; Patients aged 70 years and above received 100 mg per dose, three times daily. Treatment continued for three consecutive months. The observation group received the same treatment as the control group, with the addition of HXTQD, which is indicated for invigorating blood and unblocking the orifices. The decoction comprises the following ingredients: Huangqi (*Astragalus membranaceus*) 25 g, Fuling (*Poria cocos*), Baizhu (*Atractylodes macrocephala*) and Taizishen (*Pseudostellaria heterophylla*), 15 g each; Chuanxiong (*Ligusticum chuanxiong*) 12 g; Fa Banxia (*Pinelliae praeparatum*), Zhuru (*Bambusae caulis in taeniam*), Chishao (*Paeonia rubra*), Yizhiren (*Alpinia oxyphylla*) and Taoren (*Persicae semen*), 10 g each; Shichangpu (*Acorus tatarinowii*) and Zhishi (*Aurantii fructus immaturus*), 9 g each; Juhong (*Citrus reticulata*), Yuanzhi (*Polygala tenuifolia*) and Gancao (*Glycyrrhiza*), 6 g each; Quanxie (*Scorpio*) and Wugong (*Scolopendra*), 3 g each. The decoction was prepared in the hospital’s standardized decoction facility following traditional methods. The herbs were soaked for 30 minutes and then decocted using an automatic herbal extractor. A total of 400 mL of decoction was produced per day, divided into two 200 mL doses and taken warm 30 minutes after breakfast and dinner. The treatment lasted for three consecutive months.

### Outcome measures:

### TCM Syndrome Score:

According to the differentiation scale for the intertwined phlegm and blood stasis syndrome in the *Guiding Principles for Clinical Research of New Chinese Medicines*^4^, four core symptoms were assessed, including cognitive decline, head heaviness and headache, dark or purplish lips and nails and excessive sputum or drooling. Each symptom was graded on a 7-point scale: none (0 points), mild (2 points), moderate (4 points) and severe (six points). An increase in post-treatment score indicated symptom aggravation, while a decrease signified improvement.

### MoCA Score:

Seven cognitive domains were assessed, including visuospatial and executive functions (5 points), naming (three points), attention (six points), language (six points), abstraction (two points), delayed recall (five points) and orientation (six points), with a possible total score of 30. Higher scores indicate better cognitive function.

### MMSE Score:

The severity of cognitive impairment was evaluated using the MMSE scale, which encompasses five domains: orientation (10 points), attention and calculation (five points), memory (three points), recall (three points) and language ability (nine points). The possible total score is 30, with higher scores indicating milder cognitive impairment.

### Activities of Daily Living(ADL) Scale:

Basic self-care abilities, including eating, bathing, dressing, toileting and other daily activities were assessed, with a maximum score of 100. Functional impairment is categorized as follows:

- Mild impairment: >60 points;
- Moderate impairment: 41-60 points;
- Severe impairment: ≤40 points.

### Cerebral Hemodynamic Parameters:

Transcranial Doppler (TCD) ultrasonography was performed before and after treatment to evaluate cerebral blood flow. The following parameters of the bilateral middle cerebral arteries were recorded: mean flow velocity (Vm), resistance index (RI) and pulsatility index (PI).

### Serum Biochemical Markers:

Peripheral venous blood (4 mL) was collected from each patient in the early morning before and after treatment. Serum was separated by centrifugation. Superoxide dismutase (SOD) and glutathione peroxidase (GSH-Px) levels were measured using enzyme-linked immunosorbent assay. Nitric oxide (NO) levels were determined using chemiluminescence detection.

### Safety Assessment:

Routine examinations were conducted one day before treatment initiation and after three months of treatment. These included a fecal occult blood test, urinalysis, a complete blood count, comprehensive metabolic panel, electrocardiography and a serum electrolyte test to monitor vital signs and systemic health. Any adverse drug reactions (ADRs), such as gastrointestinal discomfort, rash, appetite loss, drowsiness, or insomnia, were documented throughout the treatment period in both groups.

### Criteria for Clinical Efficacy Evaluation^5^:

Clinical efficacy was classified into four categories:

- Complete response (CR): Complete resolution of clinical symptoms, full recovery of cognitive function and ≥95% reduction in TCM syndrome score;
- Significant response (SR): Significant improvement in clinical symptoms, cognitive function largely restored and 70%-94% reduction in TCM syndrome score;
- Partial response (PR): Partial relief of clinical symptoms with residual cognitive impairment and a 30%-69% reduction in TCM syndrome score;
- No response (NR): No improvement in clinical symptoms or cognitive function, with <30% reduction in TCM syndrome score. Overall response rate (ORR) =(Number of CR + SR + PR cases) / Total number of cases ×100%.

### Statistical analysis:

All statistical analyses were performed using SPSS24.0. Measurement data were expressed as mean ± standard deviation (*χ̅*±*S*) and t-tests were used for between-group comparisons. Categorical data were expressed as *n* (%) and analyzed using the chi-square (χ²) test. A two-tailed P-value < 0.05 was considered statistically significant.

## RESULTS

After treatment, scores for the symptoms of cognitive decline, head heaviness and headache, dark or purplish lips and nails and excessive sputum or drooling significantly decreased in both groups (all *P <* 0.05), with the observation group showing significantly lower post-treatment scores than those in the control group (all *P <* 0.05) (Table-I).

**Table-I:** Comparison of TCM syndrome scores between groups (*χ̅*±*S*).

Group	n	Cognitive Decline		Head Heaviness and Headache		Dark or Purplish Lips and Nails		Excessive Sputum or Drooling	
		Pre-treatment	Post-treatment	Pre-treatment	Post-treatment	Pre-treatment	Post-treatment	Pre-treatment	Post-treatment
Observation	40	4.02±0.40	1.15±0.16*^#^	4.05±0.41	0.93±0.10*^#^	4.11±0.38	0.93±0.08*^#^	4.16±0.37	0.91±0.08*^#^
Control	40	4.09±0.39	1.82±0.25*	4.11±0.43	1.78±0.23*	4.17±0.40	1.69±0.12*	4.12±0.39	1.67±0.20*

***Note:*** Compared with pre-treatment scores,

* *P* < 0.05; compared with the control group,

# *P* < 0.05.

Following treatment, MoCA, MMSE and ADL scores significantly increased in both groups (*P* < 0.05, respectively), with the observation group showing significantly greater improvements compared with the control group (*P* < 0.05, respectively) (Table-II).

**Table-II:** Comparison of MoCA, MMSE and ADL scores between groups (*χ̅*±*S*).

Group	n	MoCA score		MMSE score		ADL score	
		Pre-treatment	Post-treatment	Pre-treatment	Post-treatment	Pre-treatment	Post-treatment
Observation	40	16.32±2.85	28.46±4.16*#	20.45±3.51	30.51±4.26*#	62.33±3.52	87.68±4.11*#
Control	40	16.27±2.93	23.81±3.79*	20.37±3.38	26.88±3.89*	62.37±3.55	75.05±4.09*

***Note:*** Compared with pre-treatment scores,

* *P* < 0.05; compared with the control group, ^#^*P* < 0.05.

After treatment, Vm increased significantly in both groups (both *P* < 0.05), with the observation group showing a more pronounced increase than the control group (*P* < 0.05). Meanwhile, both RI and PI decreased in both groups, with significantly greater reductions observed in the observation group (*P* < 0.05, respectively) (Table-III).

**Table-III:** Comparison of cerebral hemodynamic parameters between groups (*χ̅*±*S*).

Group	n	Vm (cm/s)		RI		PI	
		Pre-treatment	Post-treatment	Pre-treatment	Post-treatment	Pre-treatment	Post-treatment
Observation	40	53.77±6.49	61.35±7.32*#	0.59±0.08	0.50±0.05*#	0.85±0.13	0.71±0.08*#
Control	40	54.12±6.61	56.49±6.85*	0.60±0.09	0.55±0.07*	0.86±0.11	0.78±0.09*

***Note:*** Compared with pre-treatment scores,

* *P* < 0.05; compared with the control group, ^#^*P* < 0.05.

Post-treatment levels of SOD, GSH-Px and NO significantly increased in both groups (all *P* < 0.05), with the observation group exhibiting significantly higher levels than the control group (*P* < 0.05, respectively) (Table-IV). The ORR in the observation group was 92.50%, significantly higher than 72.50% in the control group (*P* < 0.05) (Table-V).

**Table-IV:** Comparison of serum biochemical markers between groups (*χ̅*±*S*).

Group	n	SOD (μU/mL)		GSH-Px (U/L)		NO (μmol/L)	
		Pre-treatment	Post-treatment	Pre-treatment	Post-treatment	Pre-treatment	Post-treatment
Observation	40	156.27±27.35	287.49±41.15*#	48.69±5.14	76.13±8.80*#	2.02±0.33	3.06±0.43*#
Control	40	155.89±26.97	212.67±34.73*	48.82±5.06	62.78±7.51*	2.05±0.31	2.56±0.38*

***Note:*** Compared with pre-treatment scores,

* *P* < 0.05; compared with the control group, ^#^*P* < 0.05.

**Table-V:** Comparison of clinical efficacy between groups (n[%]).

Group	n	CR	SR	PR	NR	ORR
Observation	40	9(22.50)	12(20.00)	16(40.00)	3(7.50)	37(92.50)*
Control	40	4(10.00)	11(27.50)	14(35.00)	11(27.50)	29(72.50)

***Note:*** Compared with the control group, χ² = 5.541, *P* = 0.019.

No abnormalities were observed in the fecal occult blood test, urinalysis, complete blood count, comprehensive metabolic panel, electrocardiography, or serum electrolyte test before or after treatment in either group. Furthermore, no ADR such as gastrointestinal discomfort, rash, appetite loss, drowsiness, or insomnia occurred during the treatment period in either group.

## DISCUSSION

In this clinical trial, the MoCA and the MMSE were used as primary tools for evaluating cognitive function, while the ADL scale served as a supplementary measure of functional status. The results revealed that post-treatment MoCA, MMSE and ADL scores were significantly higher in the observation group than in the control group (all *P* < 0.05), indicating that the combination of HXTQD and aniracetam can effectively improve cognitive function and daily living ability in elderly patients recovering from ischemic stroke. These improvements suggest a potential to enhance patients’ overall quality of life.

In terms of TCM syndromes, the observation group showed significantly lower scores for cognitive decline, head heaviness and headache, dark or purplish lips and nails and excessive sputum or drooling compared with the control group (all *P* < 0.05). Additionally, the observation group achieved a higher ORR than the control group (*P* < 0.05). These findings suggest that HXTQD plus aniracetam can effectively alleviate the TCM symptoms associated with the intertwined phlegm and blood stasis syndrome in elderly patients with PISCI, thereby enhancing clinical efficacy. These benefits are closely related to HXTQD’s actions in resolving phlegm and stasis, invigorating blood, unblocking the collaterals, awakening the spirit and opening the orifices. TCD ultrasonography provides a noninvasive and dynamic assessment of cerebral vasculature. Specifically, Vm is considered a core parameter in TCD spectral analysis, while RI and PI reflect vascular tone and compliance, respectively. The middle cerebral artery (MCA) is one of the most critical arteries involved in cerebral circulation and is commonly affected following ischemic stroke. Thus, tracking changes in Vm, RI and PI of the MCA using TCD ultrasonography serves as an effective means of evaluating cerebral blood flow and cerebrovascular elasticity in patients with PISCI. In this study, Vm was significantly higher and RI and PI were significantly lower in the observation group compared with the control group after treatment (*P* < 0.05, respectively), indicating that the combination therapy can effectively regulate vascular tone and improve cerebral blood flow in elderly patients with ischemic stroke. Studies have confirmed that the onset of cognitive impairment is closely associated with chronic inflammatory responses and oxidative stress. Reactive oxygen species generated during oxidative stress can elevate lipid peroxidation, damage neuronal cells and ultimately lead to cognitive impairment.^6^ SOD is a key component of the body’s endogenous antioxidase system. A decrease in SOD levels is indicative of declining brain function and damage to cerebral tissue and vascular endothelium, which is commonly associated with impaired cognitive function.^7^ NO plays a critical role in inhibiting platelet aggregation and leukocyte adhesion, while also promoting vasodilation and enhancing cerebral blood flow regulation. Reportedly, abnormal NO expression is strongly linked to PISCI in elderly patients.^8^ GSH-Px is a widely distributed peroxidase enzyme in the human body that catalyzes the conversion of reduced glutathione to oxidized glutathione. This reaction facilitates the detoxification of harmful peroxides into harmless hydroxyl compounds, thereby protecting the structural integrity and function of cellular membranes from oxidative damage. In this study, the serum levels of SOD, GSH-Px and NO in the observation group were significantly higher than those in the control group after treatment (*P* < 0.05, respectively), indicating that HXTQD plus aniracetam can heighten the levels of these antioxidants and vasoregulatory molecules. This promotes the clearance of reactive oxygen species, inhibits the progression of cognitive impairment and contributes to the restoration of neurological and cognitive functions in affected patients. No ADRs were observed in any patients throughout the study, confirming the safety and reliability of the treatment plan.

In TCM, senile PISCI is categorized under conditions such as “*chi dai* (dementia)” or “*dai bing* (mental dullness)”, which fall within the broader domain of sequelae of stroke. According to TCM theory, the onset of stroke is often followed by obstruction of the cerebral collaterals by pathogenic factors such as phlegm-dampness and blood stasis, which leads to turbid phlegm clouding the orifices. In the elderly, these excess pathogens are compounded by deficiency of the *zang-fu* organs, particularly insufficiency of liver and kidney qi and blood, resulting in depletion of brain marrow and dysfunction of the mental faculties, ultimately manifesting as cognitive impairment or dementia. The primary pathological location of post-stroke dementia lies in the brain, but its pathogenesis is intricately connected to the five *zang* and six *fu* organs. The core pathological features involve a combination of deficiency and excess, where deficiency of the root (*ben*) coexists with excess of the branch (*biao*). The key pathological factors are deficiency, blood stasis and phlegm. The disease is chronic and progressive, with phlegm and stasis forming a mutually reinforcing cycle. Phlegm-dampness, characterized by its viscous and obstructive nature, impairs the movement of qi and blood, clouds the clear orifices and disturbs mental clarity. When it binds with other excess pathogens, it promotes the formation of blood stasis, leading to entrenched and persistent disease that is difficult to resolve.

Therefore, phlegm and blood stasis are crucial contributors to cognitive impairment and without their resolution, it’s unlikely to achieve meaningful improvement in cognitive function. In this study, HXTQD was formulated to specifically address these pathological mechanisms. The monarch herbs include Fa Banxia (*Pinelliae praeparatum*) and Zhuru (*Bambusae caulis in taeniam*), with the former drying dampness and resolving phlegm while the latter clearing heat and transforming phlegm; in combination, these herbs are particularly effective in dispersing phlegm and unblocking the orifices. The minister herbs in the formula include Fuling (*Poria cocos*), Baizhu (*Atractylodes macrocephala*), Juhong (*Citrus reticulata*), Zhishi (*Aurantii fructus immaturus*), Chuanxiong (*Ligusticum chuanxiong*), Chishao (*Paeonia rubra*) and Taoren (*Persicae semen*). Specifically, Fuling (*Poria cocos*) promotes urination, drains dampness and calms the mind; Baizhu (*Atractylodes macrocephala*) strengthens the spleen and benefits qi, with the added actions of drying dampness and promoting diuresis; Juhong (*Citrus reticulata*) resolves dampness and phlegm, regulates qi and harmonizes the middle burner; Zhishi (Aurantii fructus immaturus) dissolves phlegm, dissipates masses, breaks up *qi* stagnation and disperses accumulation; Chuanxiong (*Ligusticum chuanxiong*) activates blood circulation and promotes the movement of qi, dispels wind and relieves pain; Chishao (*Paeonia rubra*) and Taoren (*Persicae semen*) both invigorate the blood and dissolve stasis, making them suitable for treating various syndromes involving blood stasis. The assistant herbs include Huangqi (*Astragalus membranaceus*), Taizishen (*Pseudostellaria heterophylla*), Yizhiren (*Alpinia oxyphylla*), Shichangpu (*Acorus tatarinowii*), Yuanzhi (*Polygala tenuifolia*), as well as the animal-derived medicinals Quanxie (*Scorpio*) and Wugong (*Scolopendra*). Huangqi (*Astragalus membranaceus*) and Taizishen (*Pseudostellaria heterophylla*) fortify the spleen, replenish qi and invigorate qi for exterior consolidation; Yizhiren (*Alpinia oxyphylla*) warms the spleen and kidneys, arrests diarrhea, and secures essence; the combination of these three herbs effectively replenishes healthy qi, which is typically deficient in elderly patients recovering from ischemic stroke; Shichangpu (*Acorus tatarinowii*) awakens the spirit, enhances intellect, opens the orifices and dislodges phlegm; Yuanzhi (*Polygala tenuifolia*) quiets the spirit, enhances intellect, dispels phlegm and unblock the orifices. In cases of prolonged illness, disruption of qi dynamics often leads to the formation of phlegm and blood stasis, which obstructs the cerebral collaterals and clouds the orifices. This underscores the crucial importance of activating blood and unblocking the collaterals. The proposed formula is added with Quanxie (*Scorpio*) and Wugong (*Scolopendra*), which are renowned for their strong mobility and channel-entering properties. This allows the herbal constituents to reach deeply into affected areas, thereby invigorating blood, eliminating pathogenic wind, unblocking the collaterals and resolving phlegm masses.

Finally, Gancao (*Glycyrrhiza*) serves as the envoy herb, harmonizing the actions of the other ingredients and protecting the spleen and stomach from the potential harshness of the formula. Together, this formula produces a synergistic effect of resolving phlegm, dispelling stasis, invigorating blood, unblocking the collaterals, awakening the spirit and opening the orifices. Modern pharmacological studies have shown that Fa Banxia (*Pinelliae praeparatum*) exhibits significant expectorant, anti-inflammatory and cognitive-enhancing activities, as well as the ability to boost immune function.^9^ Fuling (*Poria cocos*) has neuroprotective effects and plays a modulatory role in β-amyloid production and gut microbiota management, thereby contributing to improved cognitive function in patients with Alzheimer’s disease.^10^

The active compounds of Baizhu (*Atractylodes macrocephala*) influence synaptic plasticity and enhance learning and memory functions in aging mice, suggesting therapeutic potential in the management of cognitive impairment.^11^ Zhishi (*Aurantii fructus immaturus*) has been reported to elevate levels of antioxidant enzymes such as GSH-Px and SOD and to suppress the expression of pro-inflammatory cytokines including tumor necrosis factor-α and interleukin-1β, thereby offering protection against inflammation and oxidative stress. These mechanisms play a pivotal role in the treatment of Alzheimer’s disease and the improvement of cognitive function.^12^ Tetramethylpyrazine, a major bioactive component of Chuanxiong (*Ligusticum chuanxiong*), has been shown to possess anti-inflammatory and antioxidant properties, preserve mitochondrial and synaptic function, inhibit neuronal apoptosis and support cholinergic neurotransmission, thus offering neuroprotective benefits against cognitive impairment.^13^ Chishao (*Paeonia rubra*) exhibits protective effects against cerebral ischemia-reperfusion injury and has been found to mitigate oxidative stress and inflammation while enhancing learning and memory capabilities.^14^

Taoren (*Persicae semen*) has been shown to inhibit platelet aggregation, improve hemorheological properties and provide anti-inflammatory, antioxidative, neuroprotective and neurological symptom-relieving benefits.^15^ Huangqi (*Astragalus membranaceus*) contains multiple active compounds capable of repairing cholinergic system damage, inhibiting oxidative stress, suppressing inflammatory responses and enhancing cognitive function.^16^ Yuanzhi (*Polygala tenuifolia*) has demonstrated the ability to counteract oxidative stress injury, improve mitochondrial dysfunction, reduce inflammation and inhibit acetylcholinesterase activity, all contributing to its dual role in alleviating cognitive impairment and preserving neurological function.^17^ Shichangpu (*Acorus tatarinowii*) helps improve cognitive function with its bioactive compounds that can modulate blood-brain barrier permeability, combat oxidative stress and inflammation, protect cholinergic neurons and preserve hippocampal neurons.^18^ Yuanzhi (*Polygala tenuifolia*) has been reported to inhibit tau hyperphosphorylation, block β-amyloid aggregation cascades, protect the blood-brain barrier, suppress neuronal apoptosis and ultimately enhance cognitive function.^19^

### Limitations:

However, a small number of samples is the limitation of the study. In view of this, more samples should be included in future studies to further validate the findings of this study.

## CONCLUSIONS

The combination therapy using HXTQD and aniracetam demonstrates significant clinical efficacy as it can effectively regulate the serum levels of SOD, GSH-Px and NO in elderly patients with PISCI, improve cerebral perfusion, enhance cognitive function and promote neurological recovery.

### Authors’ Contributions:

**JX, XB:** Conceived, designed the study and critical analysis, and are responsible and accountable for the accuracy or integrity of the work.

**HW, JL** and **PC:** Collected the data, performed the analysis, critical review, were involved in the writing of the manuscript. All authors have read and approved the final manuscript.
